# Hybrid Binary Imperialist Competition Algorithm and Tabu Search Approach for Feature Selection Using Gene Expression Data

**DOI:** 10.1155/2016/9721713

**Published:** 2016-08-04

**Authors:** Shuaiqun Wang, Aorigele, Wei Kong, Weiming Zeng, Xiaomin Hong

**Affiliations:** ^1^Information Engineering College, Shanghai Maritime University, Shanghai 201306, China; ^2^Faculty of Engineering, University of Toyama, Toyama-shi 930-8555, Japan

## Abstract

Gene expression data composed of thousands of genes play an important role in classification platforms and disease diagnosis. Hence, it is vital to select a small subset of salient features over a large number of gene expression data. Lately, many researchers devote themselves to feature selection using diverse computational intelligence methods. However, in the progress of selecting informative genes, many computational methods face difficulties in selecting small subsets for cancer classification due to the huge number of genes (high dimension) compared to the small number of samples, noisy genes, and irrelevant genes. In this paper, we propose a new hybrid algorithm HICATS incorporating imperialist competition algorithm (ICA) which performs global search and tabu search (TS) that conducts fine-tuned search. In order to verify the performance of the proposed algorithm HICATS, we have tested it on 10 well-known benchmark gene expression classification datasets with dimensions varying from 2308 to 12600. The performance of our proposed method proved to be superior to other related works including the conventional version of binary optimization algorithm in terms of classification accuracy and the number of selected genes.

## 1. Introduction

DNA microarray technology which can measure the expression levels of thousands of genes simultaneously in the field of biological tissues and produce databases of cancer based on gene expression data [1] has great potential on cancer research. Because the conventional diagnosis method for cancer is inaccurate, gene expression data has been widely used to identify cancer biomarkers closely associated with cancer, which could be strongly complementary for the traditional histopathologic evaluation to increase the accuracy of cancer diagnosis and classification [2] and improve understanding of the pathogenesis of cancer for the discovery of new therapy. Therefore, it has gained popularity by the application of gene expression data on cancer classification, diagnosis, and treatment.

Due to the high dimensions of gene expression data compared to the small number of samples, noisy genes, and irrelevant genes, the conventional classification methods cannot be effectively applied to gene classification due to the poor classification accuracy. With the inherent property of gene data, efficient algorithms are needed to solve this problem in reasonable computational time. Therefore, many supervised machine learning algorithms, such as Bayesian networks, neural networks, and support vector machines (SVMs), combined with feature selection techniques, have been used to process the gene data [3]. Gene selection is the process of selecting the smallest subset of informative genes that are most predictive to its relative class using a classification model. The objectives of feature selection problems are maximizing the classifier ability and minimizing the gene subsets to classify samples accurately. The optimal feature selection problem from gene data is NP-hard problem. Hence, it is more effective to use metaheuristics approaches, such as nature inspired computation, to solve this problem. In recent years, metaheuristic algorithms based on global search strategy rather than local search strategy have shown their advantages in solving combinatorial optimization problems, and a number of metaheuristic approaches have been applied on feature selection, for example, genetic algorithm (GA), particle swarm optimization (PSO), tabu search (TS), and artificial bee colony (ABC).

Metaheuristic algorithms, as a kind of random search techniques, cannot guarantee finding the optimal solution every time. Due to the fact that a single metaheuristic algorithm is often trapped into an immature solution, the recent trends of research have been shifted towards the several hybrid methods. Kabir et al. [4] introduced a new hybrid genetic algorithm incorporating a local search to fine-tune the search for feature selection. Shen et al. [5] presented a hybrid PSO and TS for feature selection to improve the classification accuracy. Next, Li et al. proposed a hybrid PSO and GA [6]. Unfortunately, the experiment results did not obtain high classification accuracy. Alshamlan et al. brought out an idea of ABC to solve feature selection. They first attempted applying ABC algorithm in analyzing microarray gene expression combined with minimum redundancy maximum relevance (mRMR) [7]. Then, they also hybridized ABC and GA algorithm to select genetic feature for microarray cancer classification and the goal was to integrate the advantages of both algorithms [8]. The result obtained by ABC algorithm was improved to some extent, but the small number of genes cannot get the high accuracy. Chuang et al. [9] introduced an improved binary PSO in which the global best particle was reset to zero position when its fitness values did not change after three consecutive iterations. With a large number of selected genes, the result of the proposed algorithm obtained 100% classification accuracy in many datasets.

So, in this paper, we concentrate on imperialist competition algorithm inspired by sociopolitical behavior which is a kind of new swarm intelligent optimization algorithms to address the process of feature selection from gene expression data. It starts with an initial population and effectively searches the solution space through some specially designed operators to converge to optimal or near-optimal solution. Although ICA has been proved a potential search technique for solving optimization problem, it still faces some difficulties that ICA is easy to trap into local optima and cannot get a better result. Tabu search (TS) as a local search technique just can make up for the deficiency of the ICA algorithm. It has the ability to avoid convergence to local optimal by a flexible memory system including aspiration criterion and tabu list. Due to local search property of TS, the convergence speed of TS largely depends on the initial solution. The parallelism of population in ICA would help the TS find the promising regions of the search space very quickly. So the hybrid algorithm HICATS effectively combines the advantages of ICA and TS and shows the superiority in feature selection.

The rest of the paper is organized as follows.  Section 2 describes the related algorithm incorporating the process of generic ICA and TS.  Section 3 elaborates the proposed HICATS including the framework, individual representation, empire initialization, colonies assimilation, and fitness function evaluation.  Section 4 describes the parameter setting and the experiment result based on several benchmark gene datasets including comparative results between HICATS and other variants of PSO. Finally, concluding remarks are presented in Section 5.

## 2. Related Algorithm

### 2.1. Generic Imperialist Competition Algorithm (ICA)

**Figure 1:**
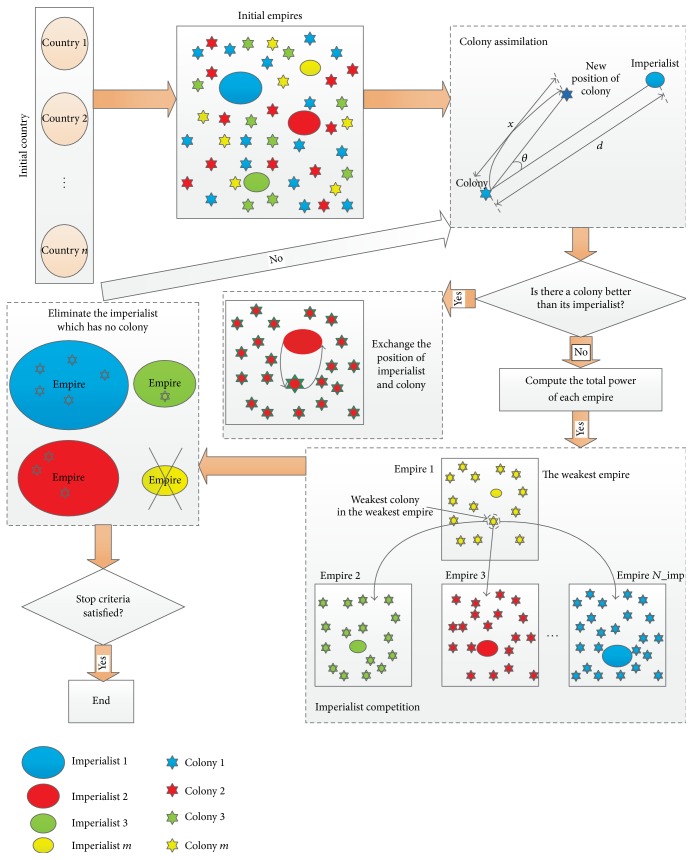
The flowchart of ICA scheme.

ICA is a population-based stochastic optimization technique, which was proposed by Atashpaz-Gargari and Lucas [10]. ICA, as one of the recent metaheuristic optimization techniques, is inspired by sociopolitical behavior. A review on last studies showed that this method has not been used to solve gene expression data for feature selection. Like other evolutionary algorithms, ICA begins with an initial set of solutions (countries) called population. Each individual of population is an array which is called “country” in ICA and “chromosome” in GA. The empire is composed of the countries that would be either an imperialist or a colony. The powerful countries are considered to be imperialists and the colonies are assigned to each empire based on the power of each imperialist state. After generating the empires, the colonies are assimilated by their related imperialist which would make the colonies stronger and move towards the promising region. If the colony is better than its imperialist when moving towards the imperialist, then exchange positions of the imperialist and its colony. As an empire has more power, it attracts more colonies and imperialist competition among the empires forms the basis of the ICA. The powerful imperialists are reinforced and the weak ones are weakened and gradually collapsed when the imperialist has no colony. Finally, the algorithm converges to the optimal solution. The flowchart of ICA is shown in Figure 1. ICA has been successfully applied in many areas: fuzzy system control, function optimization, artificial neural network training, and other application problems.

### 2.2. The Tabu Search Algorithm

Tabu search (TS) was proposed by Glober in 1986 [11], which is a famous local search algorithm, to solve a wide range of hard combination optimization problems. The algorithm begins with initial solutions and evaluates the fitness values for the given solutions. Then an associated set of feasible solutions can be obtained by applying a simple modification with given solution. This modification by a simple and basic transformation is called move. If the best of these neighbors is not in the tabu list, then select it as the new current solution. The tabu list keeps track of previously explored solutions and prevents TS from revisiting them again to avoid falling into local optimum. A move is created to increase diversity even if it is worse than the current solution. At the same time, tabu list is introduced and used to memorize the better local neighbors which have been searched and will be neglected. After a subset of feasible solutions is created according to the tabu list and evaluated by the objective function. The best optimal solution will be selected as the next solution. This loop is stopped when the stopping criteria are satisfied.

## 3. Proposed HICATS

**Figure 2:**
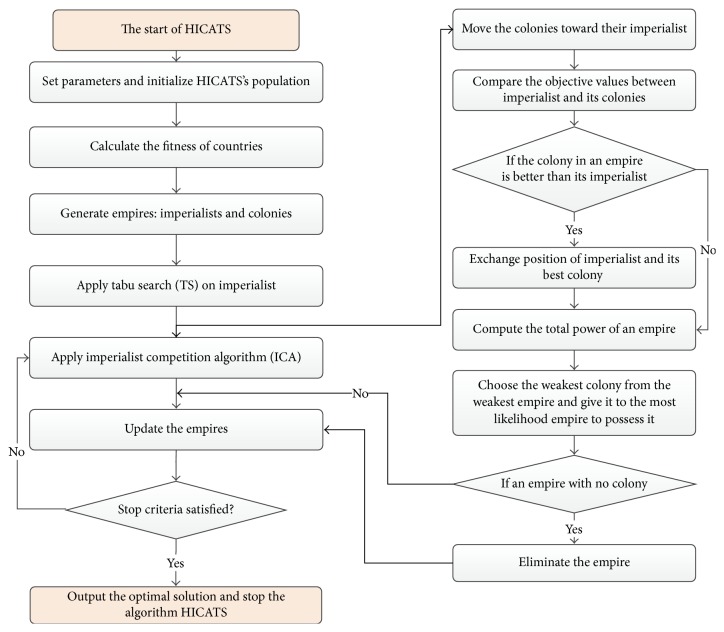
The framework of the proposed algorithm HICATS.

ICA as a global search metaheuristic algorithm reveals the advantage in solving combinatorial optimization problems; however, the diversity of the population would be greatly reduced after some generations and the algorithm might lead to premature convergence. TS as a local search technique can exploit the neighbors of current solutions to get better candidates, but it will take much time to obtain the global optimum or near-global optimum. The incorporation of TS into ICA as a local improvement strategy enables the method to maintain the population diversity and prohibits misleading local optimal. Each binary coded string (country) represents a set of genes, which is evaluated by the fitness function. TS is applied on imperialist in each empire to select the new imperialist and avoid premature convergence. The framework of the proposed algorithm HICATS can be shown in Figure 2, which is described further as follows.

**Step 1 .**

Set parameters of the algorithm and initialize countries with binary representation 0 and 1. Evaluate each country in the population which utilizes support vector machine classifier (SVM). The fitness is decided by the percentage of classification accuracy of SVM and the number of feature subsets. Then empires are generated depending on their fitness values.

**Step 2 .**

Apply TS on imperialist in each empire. Generate and evaluate the neighbors of imperialist. Select the new solution according to the tabu list and aspiration criterion to replace the old imperialist.

**Step 3 .**

Apply a learning mechanism on colonies which is the same as Baldwinian Learning (BL) mechanism [12]. Find out the different genes between imperialist and one of its colonies; then use the randomly generated learning probability to decide the number of selected genes for a colony. This strategy makes the colonies move towards their imperialist.

**Step 4 .**

Compare the objective values between imperialist and its colonies in the same empire. Exchange the positions of imperialist and its colony when a colony is better than its imperialist.

**Step 5 .**

Calculate the total power of an empire and compare all empires; then eliminate the weakest empire when it loses all of its colonies.

**Step 6 .**

If the termination condition (the predefined max iterations) is not fulfilled, go back to Step 2. Otherwise, output the optimal solution in the current population and stop the algorithm.

It is clear that HICATS integrates two quite different search strategies for feature selection, that is, the operation on ICA which can explore the new region and provide the ideal solution for TS, while TS can exploit the neighbors of imperialist for better candidate and avoid getting into local optimal according to memory system. The evaluation function, incorporating the accuracy of SVM with the number of selected genes in feature subset, assists HICATS to find the most salient features with less redundancy. A reliable selection method for genes classification should have higher classification accuracy and contain less redundant genes. For more comprehensibility, details about each component of HICATS are described in the following sections.

### 3.1. Individual Representation and Empire Initialization

**Figure 3:**
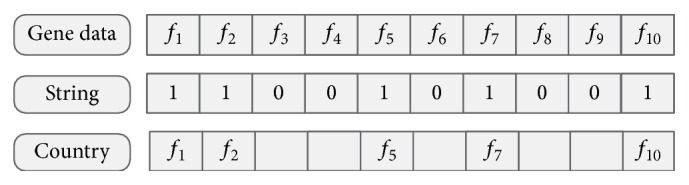
An illustrated example with generated subset and individual representation.

In this paper, we utilize random approach to generate a binary coded string (country) composed of 0, 1 and its length is equal to the dimensions of gene expression data. A value of 1 in country indicates that this gene should be selected while the value of 0 represents the uselessness of corresponding gene. In order to clearly understand these operations, we take an example for explanation. Assume that the gene data have 10 dimensions (10 features: *f*_1_, *f*_2_, *f*_3_, *f*_4_, *f*_5_, *f*_6_, *f*_7_, *f*_8_, *f*_9_, and *f*_10_); the country *X*_country_ = {1,1, 0,0, 1,0, 1,0, 0,1} is initialized with 0 and 1. The bits of the string template are 10 which is equal to the dimensions of gene data. The string is randomly generated and the number of 1 is 5. Hence, the features *f*_1_, *f*_2_, *f*_5_, *f*_7_, and *f*_10_ are selected to form a country which is shown in Figure 3.

After generating the population, we should evaluate the countries and initialize empires composed of imperialists and colonies. The fitness value of a country is estimated by the fitness function *F*:

(1) $$\begin{array}{c} fit=F\left(\;X_{country}\;\right)=F\left(\;f_{1},f_{2},\ldots ,f_{D}\;\right). \end{array}$$

In this study, assume that the initial population size is *N*_pop_; *N*_imp_ most powerful countries are selected as imperialists and the remaining *N*_col_  (*N*_col_ = *N*_pop_ − *N*_imp_) countries are assigned to these empires according to the power of imperialists as their colonies. To assign the colonies among imperialists proportionally, normalized fitness value of *m*th imperialist is defined by

(2) $$\begin{array}{c} Fit_{m}=fit_{m}-\min\left\{\;fit_{i}\;\right\},\;i\in 1,2,\ldots ,N_{imp}, \end{array}$$

where Fit_*m*_ and fit_*m*_ are the normalized fitness value of *m*th imperialist and the fitness value of *m*th imperialist, respectively. The normalized power for this imperialist is defined by

(3) $$\begin{array}{c} p_{m}=\left|\;\frac{Fit_{m}}{\sum_{i=1}^{N_{imp}}Fit_{i}}\;\right|. \end{array}$$

The normalized power of an imperialist reveals the strength of this imperialist. So the possessed colonies of *m*th empire will be

(4) $$\begin{array}{c} NC_{mcol}=round\left\{\;p_{m}\cdot N_{col}\;\right\}, \end{array}$$

where *N*_col_ is the total number of colonies and *NC*_*m*col_ is the initial number of colonies of *m*th empire. To generate each empire, we randomly choose *NC*_*m*col_ colonies and give them to each imperialist. Figure 1 shows the initial population of each empire including imperialist and colonies with the same color. It is obvious that bigger imperialists have greater number of colonies while weaker ones have less. Imperialist 1 has the most colonies and formed the most powerful empire.

### 3.2. Colonies Assimilation

In HICATS, assimilation is an important operation and could likely be a momentous help in the progress of colonies evolution. In this paper, the idea of continuous BL is introduced into the HICATS for colonies assimilation by their imperialist. This strategy can utilize some specific differential information from the imperialist, that is, the differential information between imperialist and colony *X*_IM_ − *X*_CO_, indicating a more effective way to learn from the excellent solution. It can be defined as follows:

(5) $$\begin{array}{c} X_{CO}=X_{CO}+\left\lfloor \;\beta *\left(\;X_{IM}-X_{CO}\;\right)\;\right\rfloor . \end{array}$$

**Figure 4:**
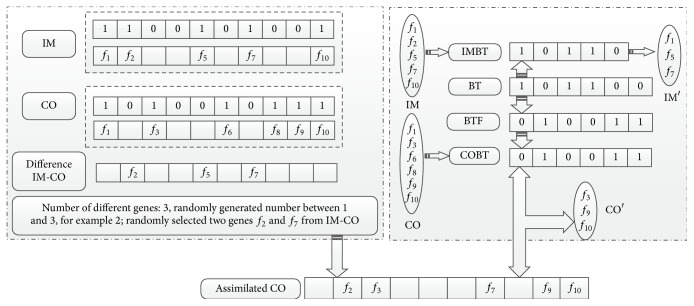
A colony is assimilated by an imperialist.

The operation of difference states that, 1 subtracting 0, the result is 1; 1 subtracting 1, the result is 0; 0 subtracting 1, the result is 0; and 0 subtracting 0, the result is 0. The learning rate *β* ∈ (0,1) is a randomly generated real number which means the proportion of selected genes from the differences. ⌊·⌋ is the operator that rounds its argument towards the closest integer and ⌊*β∗*(*X*_IM_ − *X*_CO_)⌋ represents the selected genes. In order to reduce the dimension of the country, our research adopts a randomly generated template depending on the larger dimensions of imperialist and colonies. For imperialist (IM) with five characteristics *f*_1_, *f*_2_, *f*_5_, *f*_7_, and *f*_10_ and one of its colonies with six characteristics *f*_1_, *f*_3_, *f*_6_, *f*_8_, *f*_9_, and *f*_10_ in an empire, the dimension of binary template (BT) is 6. The template of colony is generated from nonoperation of BT, denoted by BTF. Because the number of IM feature genes is less than the template BT, the IMBT just takes one part of BT. To describe how it works, we will illustrate the following numerical example. Imperialist represents *X*_IM_ = {1,1, 0,0, 1,0, 1,0, 0,1}; one of its colonies encodes *X*_CO_ = {1,0, 1,0, 0,1, 0,1, 1,1}; then the differential information is described as *X*_IM_ − *X*_CO_ = {0,1, 0,0, 1,0, 1,0, 0,0}. It is obvious that the number of different genes is 3 (the number of 1). According to the parameter *β*, the number of selected genes from different genes set is 2 and the features of *f*_2_ and *f*_7_ are chosen. At the same time, BT = {1,0, 1,1, 0,0} is produced by random strategy and BTF = {0,1, 0,0, 1,1} is the nonoperation of BT. IMBT = {1,0, 1,1, 0} is obtained from BT and COBT = {0,1, 0,0, 1,1} is equal to BTF. After this process, CO becomes a country with the features *f*_3_, *f*_9_, and *f*_10_ and the assimilated CO combining different genes between CO and IM, with five features *f*_2_, *f*_3_, *f*_7_, *f*_9_, and *f*_10_, is produced. Therefore, assimilated CO is generated by BL operation which is shown in Figure 4.

### 3.3. Fitness Function

The feature selection of gene expression data needs to consider the classification accuracy and the number of selected informative genes. Hence, the fitness function is defined as follows:

(6) $$\begin{array}{c} fitness\left(\;X_{i}\;\right)=w_{1}\times A\left(\;X_{i}\;\right)+\left(\;w_{2}\times \frac{n-D\left(X_{i}\right)}{n}\;\right) \end{array}$$

in which *A*(*X*_*i*_)∈[0,1] is the leave-one-out-cross-validation (LOOCV) classification accuracy of one country *X*_*i*_ (gene subset) obtained by SVM model. *n* is the dimensions of optimal problem; in other words, it is the total number of genes for each sample and *D*(*X*_*i*_) is the number of selected genes in *X*_*i*_. We use the parameters *w*_1_ and *w*_2_ to measure the importance of classification accuracy and the number of selected genes, respectively. The classification accuracy is more crucial than the number of selected genes and setting of the parameters satisfies constraint condition as follows: *w*_1_ ∈ [0,1] and *w*_2_ = 1 − *w*_1_.

### 3.4. TS-Based Local Search

**Figure 5:**
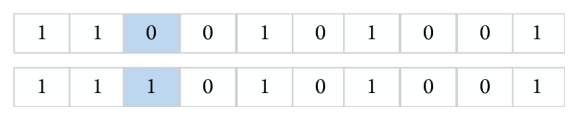
Producing nearby solutions in TS.

In HICATS, each colony can be assimilated by its imperialist and then improve itself. Thus, the whole algorithm has a speed convergence. However, the classical ICA is easy to fall into local optimum. Therefore, the exploitation is performed by TS to search the better solution nearby the current imperialist and to escape from local optimal in this paper. How to produce the solution and the tabu list is very important in TS algorithm. In our study, one bit of the solution with nonoperation is utilized to produce the nearby solutions. For example, if the gene expression data with 10 dimensions and the current country is *X*_country_ = {1,1, 0,0, 1,0, 1,0, 0,1}, the nearby solution can be obtained from the current solution through TS-based algorithm in Figure 5.

## 4. Experiment

### 4.1. Gene Expression Datasets and Parameter Setting

In this paper, except for SRBCT which was gained by continuous image analysis, the rest of the gene microarray datasets were obtained by the oligonucleotide technique. Presently, there is no standard method for processing gene expression data. Therefore, we designed an effective algorithm HICATS to perform feature selection for improving the classification accuracy. The datasets consist of 10 pieces of gene expression data, which can be downloaded from http://www.gems-system.org/. The description of datasets is listed in Table 1, which contains the dataset name and detailed expression.  Table 2 gives the related samples, genes, and classes. These datasets contained binary-class and multiclass data that contained thousands of genes.

The parameter values for HICATS are shown in Table 3. It is very clear that the parameters of the proposed algorithm are less than binary particle swarm optimization (BPSO). Hence, the influence of parameter setting on HICATS is relatively small and the robustness of algorithm is better. The size of the population affects the performance of the algorithm and computation efficiency. Large number of countries would require more computational times for completing feature selection while if the number is too small, although the algorithm can take place in a relatively short period of time, the performance of the algorithm is not guaranteed. Therefore, the intermediate values for the size of population and iteration are chosen to be 15 and 50, respectively. Since population is composed of imperialists and colonies, the number of imperialists also needs to be determined. If the number of imperialists is 1, the HICATS algorithm is transformed to single-population evolutionary algorithm instead of multisubpopulation while if the number of imperialists is too large, the number of colonies cannot be guaranteed. The number of imperialists is chosen to be 4 in our experiment. In Section 3.3, the parameters of *w*_1_ and *w*_2_ are introduced and the range of values is given. In order to guarantee that *w*_1_ is larger than *w*_2_, the values of *w*_1_ and *w*_2_ are set as 0.8 and 0.2 in our proposed algorithm with the same parameter setting of EPSO [13].

### 4.2. Experiment Results

In this paper, a hybrid algorithm HICATS incorporating ICA and TS is used to perform feature selection for the gene expression data. TS was embedded in the ICA to prevent the method from getting trapped into a local optimum, while applying TS on imperialist can improve the performance and speed up the convergence of TS.

The experiment results included classification accuracy and the number of selected feature genes obtained by HICATS over 10 independent runs for 10 datasets included 11_Tumors, 9_Tumors, SRBCT, Leukemia 1, Leukemia 2, DLBCL, Prostate_Tumor, Lung_Cancer, Brain_Tumors 1, and Brain_Tumors 2 which are shown in Tables 4, 5, and 6. It is found that the classification accuracy of HICATS achieves 100% with less than 10 informative genes for Leukemia 1, Leukemia 2, and DLBCL and with less than 20 selected genes for SRBCT. The average classification accuracy is more than 92.22% for all the datasets except for 9_Tumors. In other words, it is strongly demonstrated that HICATS can efficiently select informative genes from high-dimensional, binary-class, or multiclass datasets for classification. For all best classification results, the selected genes are less than 10 except for 9_Tumors and 11_Tumors, while, for the average classification result, the informative genes in subset are also less than 10 except for 9_Tumors, 11_Tumors, and SRBCT datasets. Furthermore, the standard deviation is less than 5 for all datasets except for 9_Tumors and 11_Tumors. From the classification accuracy and the selected informative genes, it is not difficult to find that HICATS is an efficient algorithm for feature selection and produces a near-optimal gene subset from gene expression data.

In order to verify the effectiveness of the proposed algorithm, firstly, we will compare the performance of HICATS with pure ICA using SVM as a classifier under the same experimental conditions; then, we will compare HICATS with other optimization algorithms on several benchmark classification datasets. The comparison results including the optimal classification accuracy and the number of selected genes obtained by HICATS and ICA are given in Table 7. The difference between these two algorithms is only whether each contains local search mechanism TS or not. It is quite clear that HICATS performs better than original ICA for all datasets. Hence, ICA combined with TS can effectively jump out of local optimum and HICATS achieves better performance.  Table 8 compares experiment results obtained by other approaches from the literature and the proposed method HICATS. Various methods including non-SVM and MC-SVM were used to compare with our proposed method. The experiment results listed in Table 8 were taken from Chuang et al. for comparison [9]. Non-SVM contains the *K*-nearest neighbor method [9, 14, 15], backpropagation neural networks [16], and probabilistic neural networks [17]. MC-SVM includes one-versus-one and one-versus-the-rest [18], DAG-SVM [19], the method by Weston and Watkins [20, 21], and the method by Crammer and Singer [20, 22]. It is obvious that our proposed approach HICATS obtained all the highest classification accuracies for the 10 benchmark datasets. The average highest classification accuracy of non-SVM, MC-SVM, and HICATS is 97.14, 93.63, and 97.81, respectively. For the datasets of Leukemia 1, Leukemia 2, SRBCT, and DLBCL, the classification accuracy can reach 100%. The average classification accuracy of HICATS and IBPSO seems to be the same; however, the selected genes of HICATS are significantly less than those of IBPSO listed in Table 9 because dimension reduction mechanisms are different. IBPSO algorithm mainly utilizes the value of sigmoid function to determine whether the gene is selected. In the initial iteration, the probabilities of 0 and 1 are 0.5 by a standard sigmoid function without any constraint and no modification. Then, in the next iteration, the probabilities are potentially influenced by velocity vectors; however, the probabilities of 0 and 1 are mostly maintained for its application on the gene expression data due to its high dimensions and a large search space. The number of genes is minimized about half of the total number of genes using the standard sigmoid function in high-dimensional data. Therefore, Mohamad et al. [13] introduced a modified sigmoid function to increase the probability of the bits in a particle's position to be zero and minimized the number of selected genes. In our proposed algorithm, randomly generated binary templates are used to reduce the dimension of selected genes in each generation due to the assimilation mechanism that the colonies learn a lot of different genes from their imperialist. Hence, it is not hard to find that the speed of convergence is very fast and the differences of the number of selected genes between HICATS and IBPSO are huge.

**Figure 6:**
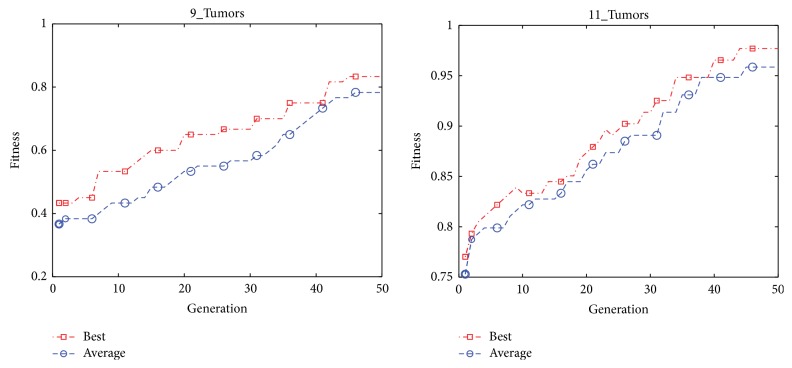
The convergence graphs of the best and average accuracy classification by HICATS algorithm on 9_Tumors and 11_Tumors datasets.

**Figure 7:**
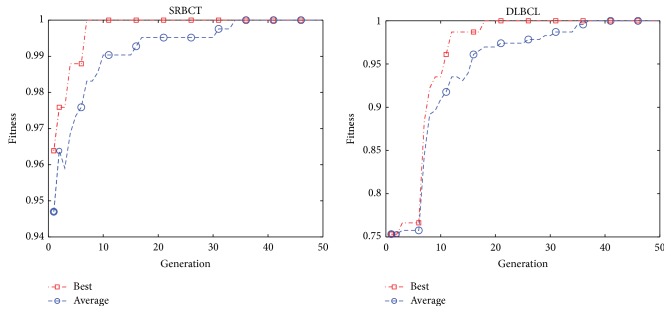
The convergence graphs of the best and average accuracy classification by HICATS algorithm on SRBCT and DLBCL datasets.

The convergence graphs of the best and average classification accuracy obtained by HICATS for 9_Tumors, 11_Tumors, SRBCT, and DLBCL are shown in Figures 6 and 7. It can be seen that the best classification accuracy is achieved to be 100% less than 10 iterations for SRBCT and between 10 and 20 generations for DLBCL. Therefore, HICATS possesses a faster convergence speed and achieves the optimal solution rapidly.

## 5. Conclusions

In this paper, a hybrid algorithm HICATS incorporated binary imperialist competition algorithm and tabu search is used to perform feature selection and SVM with one-versus-the-rest serves as an evaluator of HICATS for gene expression data classification problems. This work effectively combines the advantages of two kinds of different search mechanism algorithms to obtain the higher classification accuracy for gene expression data problems. In general, the classification performance of HICATS is as good as IBPSO; however, HICATS is superior to IBPSO and other methods in terms of selected genes. In our proposed algorithm, in order to avoid imperialist premature convergence, a local search strategy TS embedded in ICA while TS is applied on imperialist in each empire can exploit the neighbors of imperialist to speed the convergence and assist in the imperialist evolution. Experimental results show that our method effectively classifies the samples with reduced feature genes. In the future work, imperialist competition algorithm combined with other intelligent search strategies will be used to select informative genes.

## Figures and Tables

**Table 1:** Cancer-related human gene microarray datasets used in this study.

Dataset name	Description
9_Tumors	Oligonucleotide microarray gene expression profiles for the chemosensitivity profiles of 232 chemical compounds
11_Tumors	Transcript profiles of 11 common human tumors for carcinomas of the prostate, breast, colorectum, lung, liver, gastroesophagus, pancreas, ovary, kidney, and bladder/ureter
Brain_Tumor 1	DNA microarray gene expression profiles derived from 99 patient samples. The medulloblastomas included primitive neuroectodermal tumors, atypical teratoid/rhabdoid tumors, malignant gliomas, and the medulloblastomas activated by the sonic hedgehog pathway
Brain_Tumor 2	Transcript profiles of four malignant gliomas, including classic glioblastoma, nonclassic glioblastoma, classic oligodendroglioma, and nonclassic oligodendroglioma
Leukemia 1	DNA microarray gene expression profiles of acute myelogenous leukemia (AML) and acute lymphoblastic leukemia (ALL) of B-cell and T-cell
Leukemia 2	Gene expression profiles of a chromosomal translocation to distinguish mixed-lineage leukemia, ALL, and AML
Lung_Cancer	Oligonucleotide microarray transcript profiles of 203 specimens, including lung adenocarcinomas, squamous cell lung carcinomas, pulmonary carcinomas, small-cell lung carcinomas, and normal lung tissue
SRBCT	cDNA microarray gene expression profiles of small, round blue cell tumors, which include neuroblastoma, rhabdomyosarcoma, non-Hodgkin's lymphoma, and the Ewing family of tumors
Prostate_Tumor	cDNA microarray gene expression profiles of prostate tumors. Based on MUC1 and AZGP1 gene expression, the prostate cancer can be distinguished as a subtype associated with an elevated risk of recurrence or with a decreased risk of recurrence
DLBCL	DNA microarray gene expression profiles of DLBCL, in which the DLBCL can be identified as cured versus fatal or refractory disease

**Table 2:** Description of gene expression datasets.

Dataset number	Dataset name	Number of		
		Samples	Genes	Classes
1	9_Tumors	60	5726	9
2	11_Tumors	174	12533	11
3	Brain_Tumors 1	90	5920	5
4	Brain_Tumors 2	50	10367	4
5	Leukemia 1	72	5327	3
6	Leukemia 2	72	11225	3
7	Lung_Cancer	203	12600	5
8	SRBCT	83	2308	4
9	Prostate_Tumor	102	10509	2
10	DLBCL	77	5469	2

**Table 3:** Parameter settings for HICATS.

Parameters	Values
The number of countries	15
The number of imperialists	4
The number of colonies	11
The number of iterations (generations)	50
*w*_1_	0.8
*w*_2_	0.2

**Table 4:** The computational results obtained by our proposed algorithm HICATS for 10 independent runs on 11_Tumors, 9_Tumors, and SRBCT datasets.

Runs	11_Tumors		9_Tumors		SRBCT	
	Acc. (%)	Selected genes	Acc. (%)	Selected genes	Acc. (%)	Selected genes
1	**97.70**	**287**	75.00	245	100	10
2	96.55	302	76.67	262	100	14
3	94.83	330	75.00	233	100	15
4	95.40	268	75.00	249	100	13
5	96.55	290	76.67	257	**100**	**9**
6	96.55	356	81.67	242	100	12
7	94.83	323	**83.33**	**259**	100	16
8	94.83	349	76.67	238	100	9
9	95.98	275	81.67	247	100	9
10	95.40	295	81.67	253	100	10

Ave. ± SD	95.86 ± 0.97	307.5 ± 30.46	78.33 ± 3.33	248.5 ± 9.38	100 ± 0	11.70 ± 2.67

**Table 5:** The computational results obtained by our proposed algorithm HICATS for 10 independent runs on Leukemia 1, Leukemia 2, and DLBCL datasets.

Runs	Leukemia 1		Leukemia 2		DLBCL	
	Acc. (%)	Selected genes	Acc. (%)	Selected genes	Acc. (%)	Selected genes
1	**100**	**3**	100	8	100	4
2	100	3	100	10	**100**	**3**
3	100	3	100	6	100	5
4	100	3	100	6	100	3
5	100	3	100	7	100	4
6	100	3	100	8	100	3
7	100	3	**100**	**5**	100	4
8	100	3	100	7	100	5
9	100	3	100	5	100	6
10	100	3	100	6	100	4

Ave. ± SD	100 ± 0	3 ± 0	100 ± 0	6.80 ± 1.55	100 ± 0	4.10 ± 0.99

**Table 6:** The computational results obtained by our proposed algorithm HICATS for 10 independent runs on Prostate_Tumor, Lung_Cancer, Brain_Tumor 1, and Brain_Tumor 2 datasets.

Runs	Prostate_Tumor		Lung_Cancer		Brain_Tumor 1		Brain_Tumor 2	
	Acc. (%)	Selected genes	Acc. (%)	Selected genes	Acc. (%)	Selected genes	Acc. (%)	Selected genes
1	98.04	8	95.57	6	**94.44**	**6**	94	5
2	97.06	7	96.06	6	93.33	12	90	6
3	**98.04**	**5**	96.06	9	94.44	9	94	7
4	98.04	7	95.57	8	91.11	10	92	5
5	97.06	6	96.06	7	93.33	8	92	3
6	98.04	7	97.04	11	92.22	14	94	8
7	97.06	10	96.06	8	91.11	7	92	4
8	98.04	8	96.06	7	93.33	9	**94**	**3**
9	98.04	9	96.06	9	94.44	6	90	9
10	98.04	5	**97.04**	**7**	93.33	8	94	8

Ave. ± SD	97.75 ± 0.47	7.2 ± 1.62	96.16 ± 0.50	7.8 ± 1.55	93.10 ± 1.26	8.9 ± 2.55	92.60 ± 1.60	5.8 ± 2.14

**Table 7:** Classification accuracies and selected genes obtained by HICATS and ICA for gene expression data.

Datasets	Methods			
	HICATS		ICA	
	Acc. (%)	Selected genes	Acc. (%)	Selected genes
9_Tumors	**83.33**	**259**	76.67	282
11_Tumors	**97.70**	**287**	95.98	293
Brain_Tumor 1	**94.44**	**6**	91.11	8
Brain_Tumor 2	**94**	**3**	92	5
Leukemia 1	**100**	**3**	97.50	7
Leukemia 2	**100**	**5**	97.32	8
Lung_Cancer	**97.04**	**7**	95.57	12
SRBCT	**100**	**9**	100	10
Prostate_Tumor	**98.04**	**5**	97.06	6
DLBCL	**100**	**3**	97.50	5

**Table 8:** Classification accuracies of gene expression data obtained via different classification methods.

Datasets	Methods								HICATS
	Non-SVM			MC-SVM					SVM
	*K*NN [9]	NN	PNN	OVR	OVO	DAG	WW	CS	OVR
9_Tumors	78.33	19.38	34.00	65.10	58.57	60.24	62.24	65.33	**83.33**
11_Tumors	93.10	54.14	77.21	94.68	90.36	90.36	94.68	95.30	**97.70**
Brain_Tumor 1	94.44	84.72	79.61	91.67	90.56	90.56	90.56	90.56	**94.44**
Brain_Tumor 2	94.00	60.33	62.83	77.00	77.83	77.83	73.33	72.83	**94**
Leukemia 1	100	76.61	85.00	97.50	91.32	96.07	97.50	97.50	**100**
Leukemia 2	100	91.03	83.21	97.32	95.89	95.89	95.89	95.89	**100**
Lung_Cancer	96.55	87.80	85.66	96.05	95.59	95.59	95.55	96.55	**97.04**
SRBCT	100	91.03	79.50	100	100	100	100	100	**100**
Prostate_Tumor	92.16	79.18	79.18	92.00	92.00	92.00	92.00	92.00	**98.04**
DLBCL	100	89.64	80.89	97.50	97.50	97.50	97.50	97.50	**100**

(1) Non-SVM: traditional classification method. (2) MC-SVM: multiclass support vector machines. (3) *K*NN: *K*-nearest neighbors. (4) NN: backpropagation neural networks. (5) PNN: probabilistic neural networks. (6) OVR: one-versus-the-rest. (7) OVO: one-versus-one. (8) DAG: DAGSVM. (9) WW: method by Weston and Watkins. (10) CS: method by Crammer and Singer. (11) HICATS: improved binary imperialist competition algorithm.

**Table 9:** The number of selected genes from datasets between HICATS and IBPSO.

Datasets	HICATS		IBPSO	
	Genes selected	Percentage of genes selected	Genes selected	Percentage of genes selected
9_Tumors	**259**	0.045	2941	0.51
11_Tumors	**287**	0.022	3206	0.26
Brain_Tumor 1	**6**	0.001	754	0.13
Brain_Tumor 2	**3**	0.0003	1197	0.12
Leukemia 1	**3**	0.0006	1034	0.19
Leukemia 2	**5**	0.0004	1292	0.12
Lung_Cancer	**7**	0.0005	1897	0.15
SRBCT	**9**	0.004	431	0.19
Prostate_Tumor	**5**	0.0005	1294	0.12
DLBCL	**3**	0.0005	1042	0.19

Average	**5**	0.00097	1117.6	0.15
